# Sputum microbiomic clustering in asthma and chronic obstructive pulmonary disease reveals a *Haemophilus*‐predominant subgroup

**DOI:** 10.1111/all.14058

**Published:** 2019-10-21

**Authors:** Sarah Diver, Matt Richardson, Koirobi Haldar, Michael A. Ghebre, Mohammadali Y. Ramsheh, Mona Bafadhel, Dhananjay Desai, Emma Suzanne Cohen, Paul Newbold, Laura Rapley, Paul Rugman, Ian D. Pavord, Richard D. May, Michael Barer, Christopher.E. Brightling

**Affiliations:** ^1^ Institute for Lung Health NIHR Leicester Biomedical Research Centre Department of Respiratory Sciences College of Life Sciences University of Leicester and University Hospitals of Leicester NHS Trust Leicester UK; ^2^ hVIVO Services Limited London UK; ^3^ Respiratory Medicine Unit Nuffield Department of Medicine University of Oxford Oxford UK; ^4^ AstraZeneca Cambridge UK; ^5^ AstraZeneca Gaithersburg MD USA; ^6^ GlaxoSmithKline Stevenage UK; ^7^ Sosei Heptares Cambridge UK

**Keywords:** asthma, COPD, inflammation, microbiome, sputum

## Abstract

**Background:**

Airway ecology is altered in asthma and chronic obstructive pulmonary disease (COPD). Anti‐microbial interventions might have benefit in subgroups of airway disease. Differences in sputum microbial profiles at acute exacerbation of airways disease are reflected by the γProteobacteria:Firmicutes (γP:F) ratio. We hypothesized that sputum microbiomic clusters exist in stable airways disease, which can be differentiated by the sputum γP:F ratio.

**Methods:**

Sputum samples were collected from 63 subjects with severe asthma and 78 subjects with moderate‐to‐severe COPD in a prospective single centre trial. Microbial profiles were obtained through 16S rRNA gene sequencing. Topological data analysis was used to visualize the data set and cluster analysis performed at genus level. Clinical characteristics and sputum inflammatory mediators were compared across the clusters.

**Results:**

Two ecological clusters were identified across the combined airways disease population. The smaller cluster was predominantly COPD and was characterized by dominance of *Haemophilus* at genus level (n = 20), high γP:F ratio, increased *H influenzae*, low diversity measures and increased pro‐inflammatory mediators when compared to the larger *Haemophilus*‐low cluster (n = 121), in which *Streptococcus* demonstrated the highest relative abundance at the genus level. Similar clusters were identified within disease groups individually and the γP:F ratio consistently differentiated between clusters.

**Conclusion:**

Cluster analysis by airway ecology of asthma and COPD in stable state identified two subgroups differentiated according to dominance of *Haemophilus*. The γP:F ratio was able to distinguish the *Haemophilus*‐high versus *Haemophilus*‐low subgroups, whether the *Haemophilus‐*high group might benefit from treatment strategies to modulate the airway ecology warrants further investigation.

## Graphical abstract

Sputum microbiomic cluster analysis and TDA demonstrates 2 subgroups of stable severe asthma and moderate‐to‐severe COPD differentiated according to dominance of *Haemophilus. *The *Haemophilus*‐high group had no defining clinical characteristics but had lower microbial diversity and increased levels of sputum TNFα and IL1β. γProteobacteria:Firmicutes (γP:F) differentiated the clusters and was the most predictive biomarker of the *Haemophilus*‐high group.

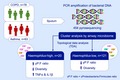

## 1 INTRODUCTION

Asthma and chronic obstructive pulmonary disease (COPD) are increasingly prevalent airways diseases responsible for considerable morbidity and resource utilization worldwide, especially in those with the most severe disease. They are well recognized to encompass a heterogeneous pathobiology, with well‐defined disease phenotypes or endotypes^1^, ^2^, ^3^ identifiable at different scales, which impact on treatment strategies for individual patients.

Bacterial colonization is an important feature in both diseases. Culture‐independent techniques based on sequencing the variable regions of bacterial 16S ribosomal RNA genes have demonstrated that the healthy airway is colonized and that the microbial composition of the airway is altered in asthma and COPD.^4^, ^5^ Associations between microbial ecology, clinical characteristics and inflammatory mediator profiles have been demonstrated, suggesting that specific pathogens may be associated with particular disease phenotypes. In particular, *Haemophilus* spp. are often the most frequently detected potential pathogens in subgroups of asthma and COPD and are associated with airway neutrophilia^6^, ^7^ and corticosteroid resistance^8^ whilst studies in childhood asthma have associated *Moraxella* with an increased risk of exacerbation.^9^

Establishing the microbial community profiles characteristic of these subgroups, at stable and exacerbation states, will assist in defining therapeutic targets. Anti‐microbial therapies have been evaluated in both asthma and COPD, as both maintenance treatment and at acute exacerbation, and are beneficial in some cases.^10^, ^11^, ^12^, ^13^ However, there are public health risks associated with widespread antibiotic use.^14^ Identification of key bacterial targets and biomarkers to distinguish those who will potentially derive a clinically significant benefit will aid clinicians to manage this risk or develop new therapies beyond antibiotics.

Previous longitudinal assessments of microbiome dynamics in COPD by our group reported a subgroup in whom dysbiosis occurred at exacerbation^15^ and proposed the use of γProteobacteria to Firmicutes (γP:F) ratio to identify this group. Furthermore, a recent analysis of biological exacerbation subgroups in asthma and COPD suggested that exacerbation aetiology varies,^16^ with a bacterial‐associated group demonstrating elevated Proteobacteria to Firmicutes ratios, compared to those observed in eosinophilic or viral‐triggered episodes. It is unclear whether separation of these subgroups is maintained at stable state.

In this study, we hypothesized that subgroups with distinct microbial profiles exist within a stable, severe asthma and moderate‐to‐severe COPD population and that the γP:F ratio can be used to distinguish these groups, potentially highlighting individuals who may benefit from therapy targeting airway dysbiosis. To test this, we examined the microbiome in sputum samples from subjects with asthma and COPD and utilized a combination of cluster analysis and topological data analysis (TDA) to define groups which could then be characterized in more detail.

## 2 METHODS

### 2.1 Study participants

Patients with severe asthma and moderate‐to‐severe COPD were prospectively recruited from a single centre at Glenfield Hospital, Leicester, UK. Asthma and COPD were diagnosed according to physician assessment consistent with definitions based on Global Initiative for Asthma (GINA) or Global Initiative for Chronic Obstructive Lung Disease (GOLD) guidance. Asthmatic subjects had participated in a published observational study (n = 131)^17^ and COPD subjects had provided stable samples, at least 8 weeks postexacerbation, whilst participating in a published longitudinal exacerbation study (n = 156).^18^ Subjects with asthma were GINA step 4 or 5, with sputum samples provided at visits when clinically stable, at least 4 weeks postexacerbation. Subjects with COPD, GOLD class I‐IV, were required to have experienced at least one exacerbation in the preceding 12 months and were excluded if they were unable to produce sputum following an induced sputum procedure, or if a current or previous diagnosis of asthma was present. Subjects requiring maintenance oral corticosteroid therapy were included in both studies. All patients provided written informed consent for samples to be used in future analyses, and all subjects with sputum samples adequate for microbiome sequencing were included in the current analysis. Both studies were approved by the local Leicestershire, Northamptonshire and Rutland ethics committee.

### 2.2 Measurements

Demographics, clinical characteristics and lung function data were collected. Severity of cough and dyspnoea was assessed using a visual analogue scale (VAS) which has previously been described.^16^ Spontaneous or induced sputum was collected for sputum total and differential cell counts and bacteriology. 95% of samples were spontaneous. Inflammatory mediator profiling was performed on cell‐free sputum supernatants using the Meso Scale Discovery Platform (MSD; Gaithersburg, MD, USA) as previously described,^19^ with values below the detectable limit replaced with the corresponding lower limit of detection prior to analysis. Bacterial load was measured by quantitative polymerase chain reaction (qPCR) with DNA extraction and qPCR performed as previously described,^18^ based on the abundance of 16S ribosomal subunit encoding genes (total 16S). Pathogen‐specific bacterial 16S abundance via qPCR was quantified for *H influenzae* using the SYBR Green assay (PE Applied Biosystems). The threshold of detection for pathogen‐specific bacterial 16S qPCR analysis was taken as 1 × 10^4^ genome copies/ml reflecting previous cut‐off thresholds used in this field.^20^ Measurements refer to genome copies/ml of homogenized sputum compared to a standard curve. For microbiomic analyses, DNA was extracted from sputum using the Qiagen DNA Mini kit (Qiagen) as per manufacturer's protocol, following which PCR‐amplification of the V4‐5 hypervariable regions of 16S ribosomal RNA were pyrosequenced on 454 Genome Sequencer FLX platform (454 Life Sciences; Roche Diagnostics) to obtain microbiome communities. Sequencing reads were processed using QIIME^21^ (quantitative insights into microbial ecology, Version 1.9.1) as previously described.^22^ Taxonomic classification, within sample (alpha‐diversity) and between sample (beta‐diversity) microbiome measures were performed at normalized sequence read depth of 1666 and 97% sequence identity. PCR reagent control and a sample with a known microbiomic profile from previous analyses were used as positive and negative controls, respectively. To ensure quality of sequencing data, low‐quality sequence reads were trimmed and adaptors, chimeras and potential human sequences were removed. γP:F ratio was calculated for each individual sample using the sequencing data, by dividing the total proportion of the sample belonging to the class Gammaproteobacteria, by the proportion belonging to the phylum Firmicutes. Sequence data are deposited at the National Center for Biotechnology Information Sequence Read Archive (SRP065072).

### 2.3 Statistical methods

Statistical analysis was performed using GraphPad Prism (Version 7) and SPSS (Version 25.0). Clustering using finite normal mixture modelling^23^ was performed using the mclust (Version 2.5.2)^24^ package in R 3.5.1 using microbiome sequencing data. Topological data analysis (TDA)^25^ was performed using KeplerMapper (Version 1.2.0),^26^ a Python implementation of the TDA Mapper algorithm,^27^ for the same data set. Parametric and nonparametric data were presented as mean (SEM) and median (inter‐quartile range) and comparisons between groups were made by unpaired *t* tests or Mann‐Whitney tests, respectively. Sensitivity and specificity of biomarkers was determined using the receiver‐operator characteristic (ROC) curve. Repeatability was assessed by the intra‐class correlation coefficient (ICC). For statistical significance, *P* < .05 was applied.

## 3 RESULTS

### 3.1 Microbiome asthma versus COPD

Clinical characteristics for subjects with severe asthma (n = 63) and moderate‐to‐severe COPD (n = 78) are as shown in Table 1. Subjects with asthma were younger and more likely to be receiving treatment with oral steroids, with a longer duration of disease and a higher BMI. Those with COPD were more likely to be male, current or ex‐smokers with a longer pack‐year history, lower lung function and were more symptomatic with cough and dyspnoea. There were no differences between disease groups in number of exacerbations in the preceding 12 months, nor neutrophils and eosinophils in blood or sputum.

**Table 1:** Clinical characteristics according to disease group

Characteristic	Asthma (n = 63)	COPD (n = 78)	*P* value
Male sex, n (%)	34 (54.0)	59 (75.6)	.007
Age^a^	55.2 (1.5)	68.0 (1.0)	<.001
Duration of disease^b^	30.0 (5.0‐49.5)	5.7 (3.7‐12.3)	<.001
BMI^a^	29.3 (0.9)	26.3 (0.5)	.004
Current/ex‐smokers, n (%)	25 (39.7)	76 (98.4)	<.001
Pack‐year history^b^, ^c^	10.0 (2.0‐18.2)	48.0 (34.9‐61.5)	<.001
Exacerbations in the last year^b^	3.0 (2.0‐4.0)	3.0 (1.0‐4.3)	.899
Maintenance prednisolone, n (%)	33 (52.4)	7 (9.0)	<.001
Daily prednisolone dose^b^, ^d^	10.0 (7.5‐12.5)	5.0 (5.0‐5.0)	.002
Daily inhaled corticosteroid dose^b^	1600 (1000‐2000)	1600 (800‐2000)	.269
Pre‐FEV_1_ (L)^a^	2.08 (0.09)	1.20 (0.06)	<.001
Pre‐FEV_1_ (% predicted)^a^	72.11 (2.70)	41.39 (1.89)	<.001
Post‐FEV_1_ (L)^a^	2.27 (0.10)	1.26 (0.06)	<.001
Post‐FEV_1_ (% predicted)^a^	78.09 (2.70)	44.00 (2.01)	<.001
FEV_1_/FVC ratio^a^	0.67 (0.02)	0.47 (0.02)	<.001
VAS, cough (mm)^b^	35.5 (9.8‐53.3)	51.0 (23.5‐72.0)	.041
VAS, dyspnoea (mm)^b^	30.0 (11.3‐53.3)	57.0 (37.5‐69.5)	<.001
Blood neutrophils (×10^9^/L)^a^	5.69 (0.27)	5.61 (0.23)	.830
Blood eosinophils (×10^9^/L)	0.26 (0.20‐0.34)	0.21 (0.18‐0.24)	.133
Sputum neutrophils count (%)^b^	67.88 (49.13‐83.44)	75.37 (59.75‐88.21)	.083
Sputum eosinophil count (%)	1.89 (1.19‐2.99)	1.20 (0.88‐1.62)	.101
γP:F ratio	0.30 (0.19‐0.47)	0.34 (0.18‐0.63)	.760

Note

Data are presented as geometric means, with 95% CI, unless otherwise stated.

Abbreviations: BMI, body mass index; COPD, chronic obstructive pulmonary disease; FEV_1_, forced expiratory volume in the first second; FVC, forced vital capacity; VAS, visual analogue scale; γP:F ratio, γProteobacteria to Firmicutes ratio.

a Mean (SEM).

b Median (1st and 3rd quartiles).

c Pack‐year history of current and ex‐smokers.

d Dose for those patients prescribed oral corticosteroids

**Figure 1:**
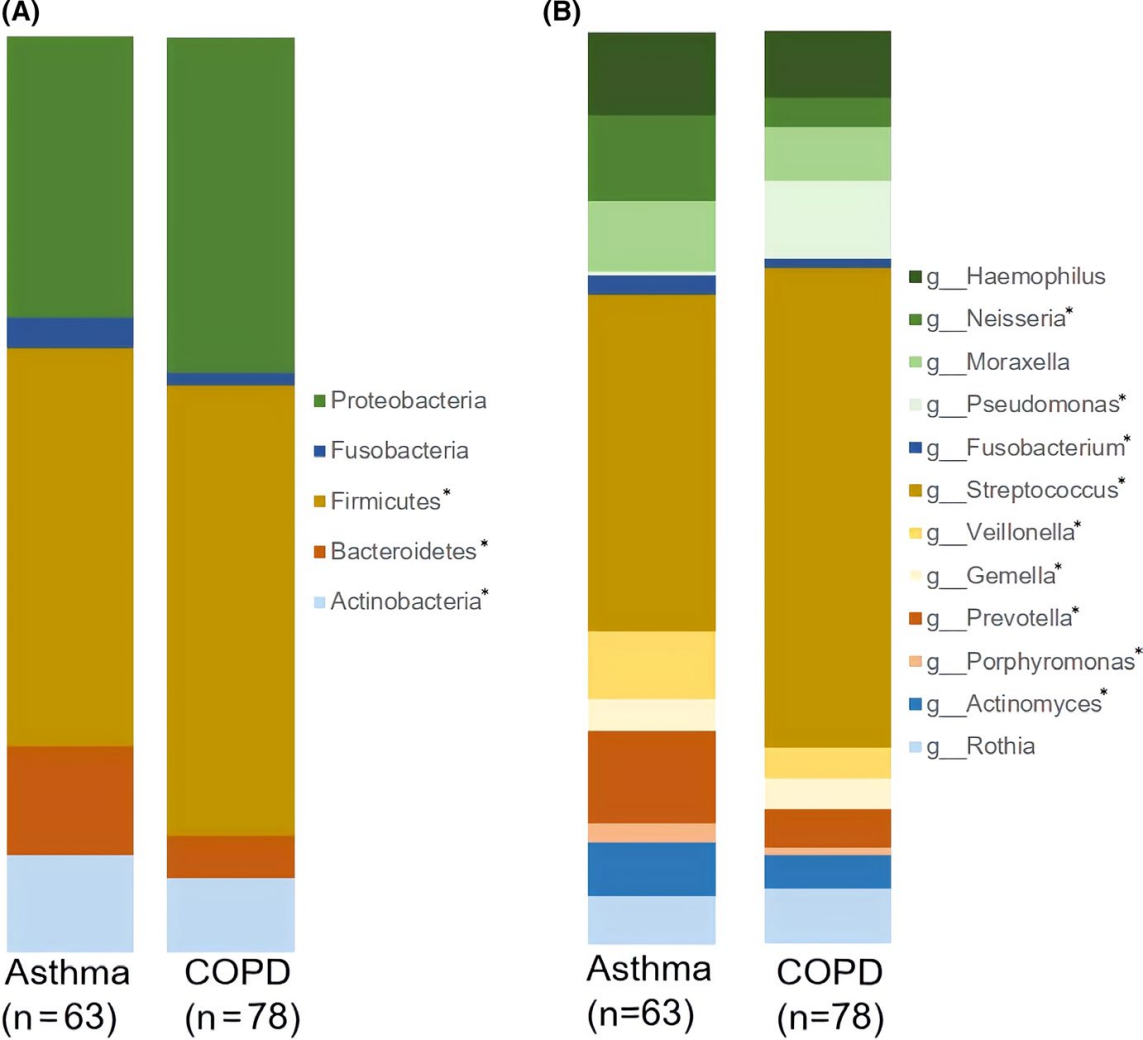
The distribution of: A, 5 most abundant phyla and B, abundant or important genera across asthma and COPD. Statistical differences between disease groups are shown with *

The distribution of the major sputum bacterial phyla and genera are shown in Figure 1A,B. At the phylum level, increased relative abundance of Actinobacteria, Bacteroidetes and Fusobacteria was observed in asthma compared to COPD; however, no difference was observed in proportions of the two most abundant phyla, Proteobacteria and Firmicutes. At the genus level, increased proportions of *Streptococcus* were observed in COPD compared to asthma; no significant differences were observed in proportions of *Haemophilus* or *Moraxella.*

COPD subjects displayed a significantly lower richness and alpha‐diversity compared to subjects with asthma (Figure S1).

### 3.2 Microbiomic clusters of stable airways disease (asthma and COPD combined)

**Figure 2:**
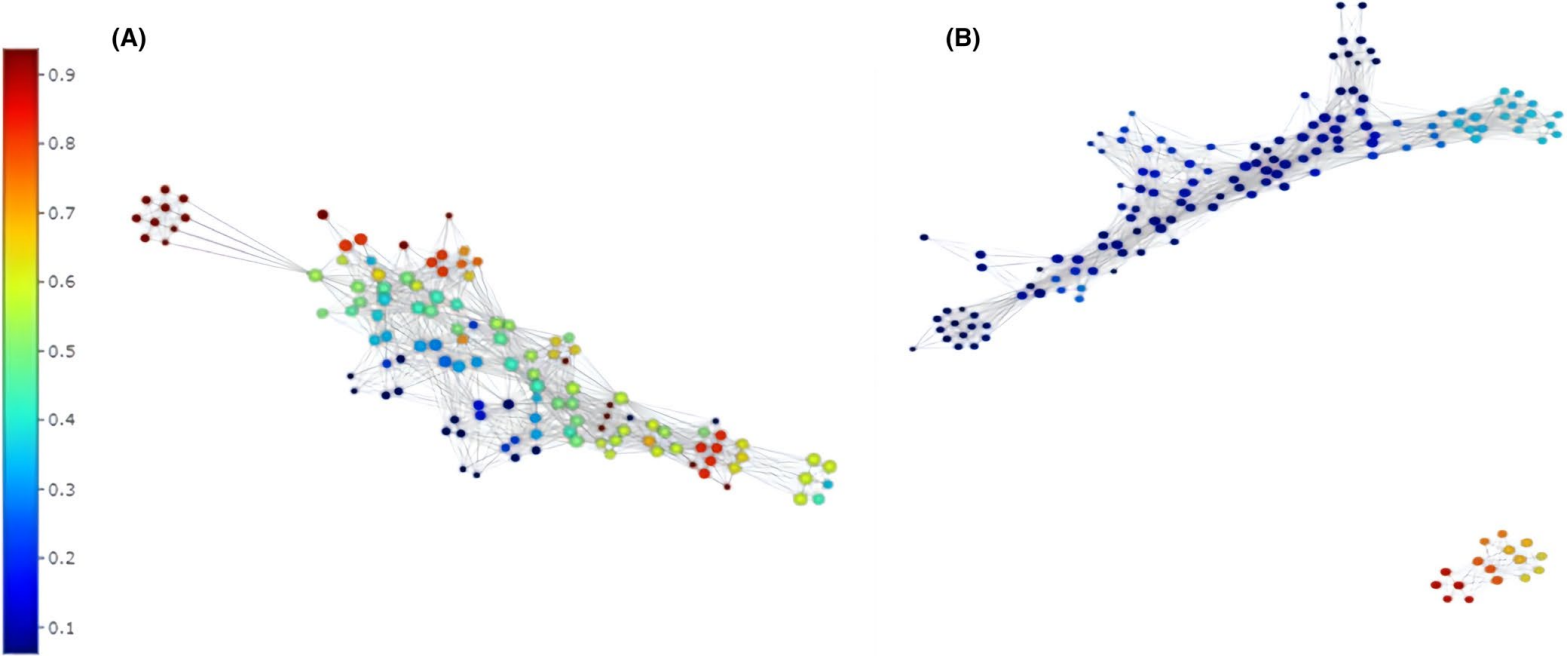
Topological data analysis for combined microbiological data (OTU proportions) from combined asthmatic and COPD subjects at: A) phylum level and B) genus level. Within each network, a single node (point) represents a sub‐cluster of subjects sharing similar proportions of OTUs. The length of the line connecting nodes represents how similar the nodes are. The longer the connecting line, the more dissimilar the nodes. At phylum level, the network was seen to be a single structure. In (A) the network is coloured by disease group; Asthma or COPD. At genus level, the network was seen to comprise two structures corresponding to two distinct clusters, (there are no connecting lines between nodes, indicating two quite distinct clusters). The networks in (B) were coloured by average OTU proportion for *Haemophilus*, red indicating high average proportions of *Haemophilus*, blue indicating low average proportions of *Haemophilus*

Proportions of the two major phyla were similar between disease groups; therefore, further analysis was performed with asthma and COPD subjects combined. Topological data analysis (TDA) was utilized to visualize the data and improve clarity regarding the presence of clusters. At the phylum level, the population appeared as a single structure with areas of differentiation. In contrast, clustering according to genus demonstrated two distinct structures that were well differentiated according to the genus *Haemophilus*, but not differentiated by other genera (Figure 2 and S2).

Cluster 1 *(Haemophilus*‐high) contained 20 subjects (14% of the total population) and was predominantly COPD (75%, compared to 52% COPD in the *Haemophilus*‐low Cluster 2). This was reflected in the clinical characteristics (Tables 2 and S1) with lower FEV_1_ demonstrated in Cluster 1 compared to Cluster 2, although no other distinguishing clinical characteristics were noted. The predominance of subjects with COPD in Cluster 1 was also reflected in an increased sputum neutrophilia compared to Cluster 2. Cluster 1 had a higher γP:F ratio across both conditions. Despite a higher proportion of asthmatics in Cluster 2, raised levels of blood and sputum eosinophilia were not observed.

**Table 2:** Clinical characteristics across the identified ecological clusters

Characteristic	Cluster 1 (Asthma = 5, COPD = 15)	Cluster 2 (Asthma = 58, COPD = 63)	*P* value
Male sex, n (%)	14 (70.0)	79 (65.3)	.680
Age^a^	66.7 (1.9)	61.6 (1.1)	.077
Duration of disease^b^	11.0 (6.3‐26.0)	13.9 (4.1‐35.0)	1.000
BMI^a^	27.6 (1.2)	27.7 (0.6)	.984
Current/ex‐smokers, n (%)	16 (80.0)	85 (70.3)	.370
Pack‐year history^b^, ^c^	36.5 (18.2‐50.7)	40.5 (16.9‐58.5)	.819
Exacerbations in the last year^b^	3.0 (1.0‐6.5)	3.0 (1.0‐4.0)	.614
Maintenance prednisolone, n (%)	4 (20.0)	36 (33.0)	.247
Daily prednisolone dose^b^, ^d^	5.0 (4.3‐5.0)	10.0 (5.0‐10.0)	.693
Daily inhaled corticosteroid dose^b^	2000 (800‐2000)	1600 (1000‐2000)	.670
Pre‐FEV_1_ (L)^a^	1.35 (0.11)	1.64 (0.07)	.036
Pre‐FEV_1_ (% predicted)^a^	46.99 (4.03)	56.71 (2.29)	.044
Post‐FEV_1_ (L)^a^	1.39 (0.12)	1.79 (0.08)	.011
Post‐FEV_1_ (% predicted)^a^	48.67 (4.50)	61.38 (2.45)	.019
FEV_1_/FVC ratio^a^	0.52 (0.03)	0.56 (0.02)	.316
VAS, cough (mm)^b^	60.5 (35.5‐80.8)	37.0 (12.0‐60.0)	.319
VAS, dyspnoea (mm)^b^	50.5 (18.0‐70.8)	40.0 (15.0‐61.0)	.336
Blood neutrophils (×10^9^/L)^a^	5.91 (0.64)	5.60 (0.17)	.644
Blood eosinophils (×10^9^/L)	0.24 (0.17‐0.33)	0.23 (0.19‐0.27)	.788
Sputum neutrophils count (%)^b^	91.05 (81.44‐95.75)	69.00 (49.81‐83.88)	.008
Sputum eosinophil count (%)	1.08 (0.57‐2.06)	1.54 (1.15‐2.06)	.346
γP:F ratio	13.19 (6.50‐26.76)	0.17 (0.12‐0.24)	<.001
Bacterial 16S (copies/mL × 10^9^)	1.51 (0.54‐4.21)	0.91 (0.62‐1.33)	.323
*H influenzae* (copies/mL × 10^9^)	1.18 (0.41‐3.45)	0.03 (0.02‐0.04)	<.001

Note

Data are presented as geometric means, with 95% CI, unless otherwise stated.

Abbreviations: BMI, body mass index; COPD, chronic obstructive pulmonary disease; FEV_1_, forced expiratory volume in the first second; FVC, forced vital capacity; VAS, visual analogue scale; γP:F ratio, γProteobacteria to Firmicutes ratio.

a Mean (SEM).

b Median (1st and 3rd quartiles).

c Pack‐year history of current and ex‐smokers.

d Dose for those patients prescribed oral corticosteroids.

**Figure 3:**
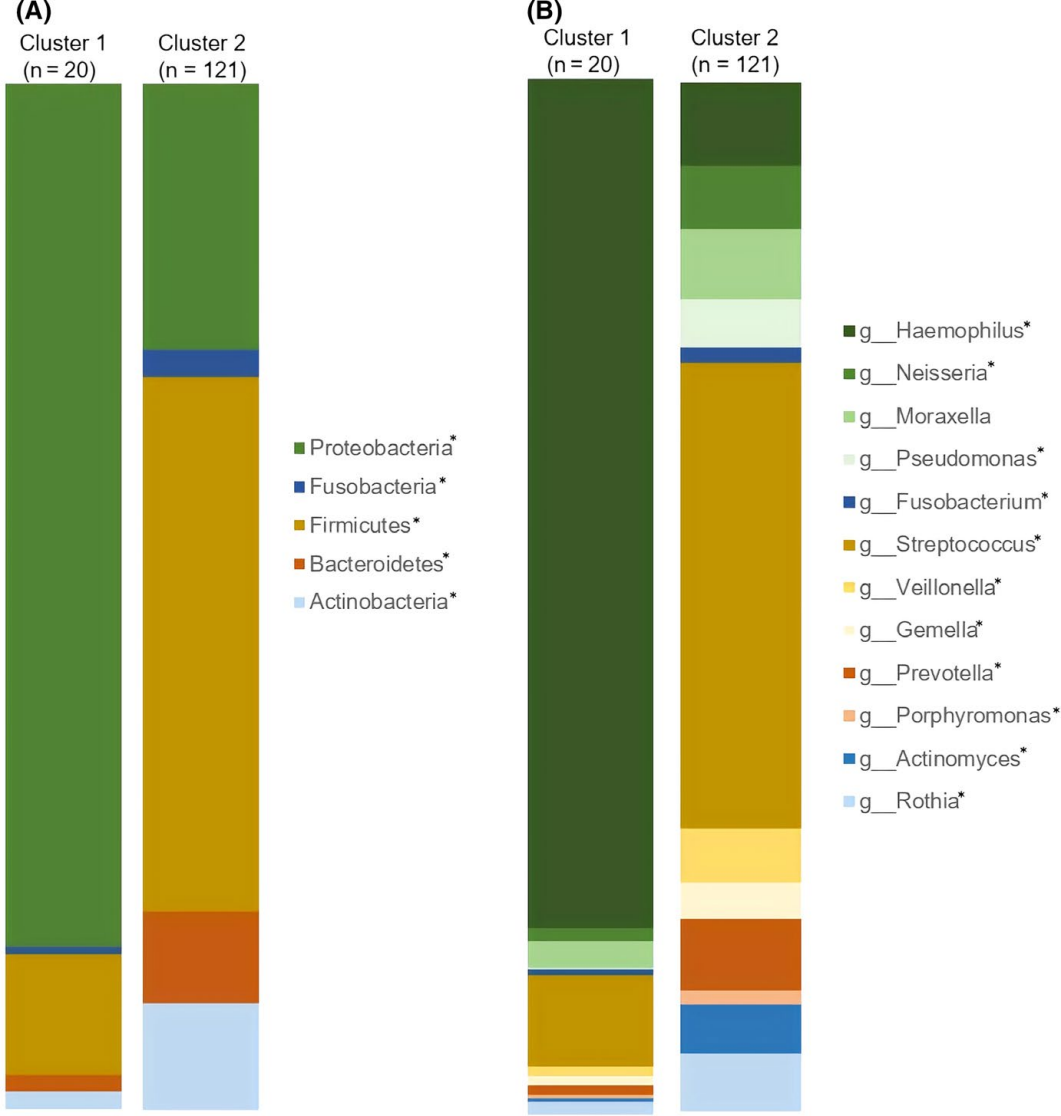
Proportions of bacteria in: A, the 5 most abundant phyla and B, abundant or important genera across the identified ecological clusters. Statistical differences between clusters are shown with *

Distribution of the major phyla and abundant or important genera are shown in Figure 3A,B. Cluster 1 had increased relative abundance of Proteobacteria compared to Cluster 2, which had increased proportions of bacteria from the phyla Firmicutes, Actinobacteria, Bacteroidetes and Fusobacteria. Of the total organisms detected from subjects in Cluster 1, 82% belonged to the genus *Haemophilus*. In Cluster 2, the most abundant genus was *Streptococcus* (45% of total).

**Figure 4:**
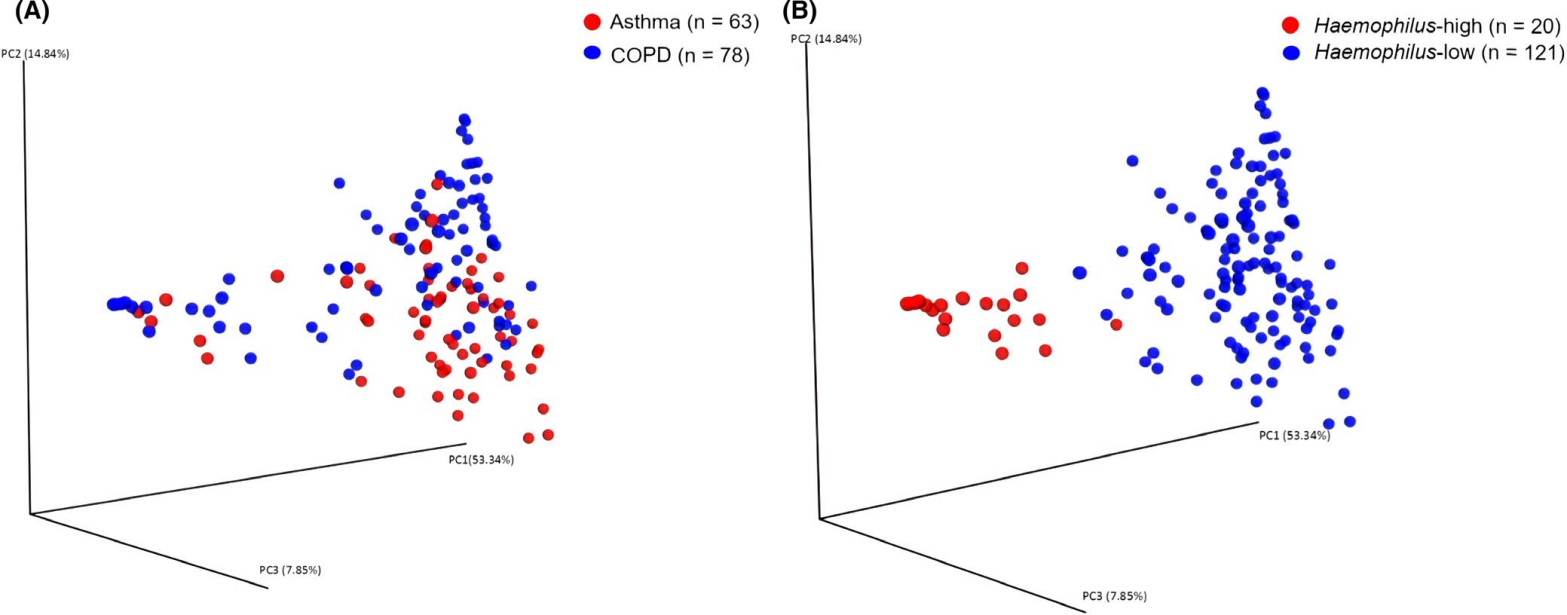
Principle components analysis plots of β‐diversity between: A, asthma and COPD disease groups and B, *Haemophilus*‐high and *Haemophilus*‐low subgroups

Subjects in the *Haemophilus*‐high group exhibited significantly lower richness and alpha‐diversity compared to the *Haemophilus*‐low group (Figure S1). Beta‐diversity analysis demonstrated a significantly higher weighted UniFrac distance between the two clusters. On comparing subjects within the respective groups, subjects within the *Haemophilus*‐high group had the lowest beta‐diversity measures. Principle component analysis on the UniFrac distance measure performed across the ecological clusters demonstrates clear separation (Figure 4), in contrast to similar analysis according to disease group which offers no clear distinction.

Sputum biological mediators were compared across the clusters (Tables 3 and S2), with the *Haemophilus*‐high cluster demonstrating higher levels of IL1β and TNFα, and lower CCL13 versus the *Haemophilus*‐low cluster.

**Table 3:** Levels of sputum mediators across the identified ecological clusters

Sputum mediator	Cluster 1 (n = 20)	Cluster 2 (n = 121)	*P* value
IL1β (pg/mL)	475.47 (170.17‐1328.52)	73.46 (54.41‐99.18)	<.001
IL5 (pg/mL)	1.78 (0.58‐5.44)	2.85 (1.93‐4.20)	.402
IL6 (pg/mL)	141.82 (30.06‐669.13)	134.39 (94.30‐191.53)	.921
IL6R (pg/mL)	257.11 (138.26‐478.13)	250.44 (204.12‐307.27)	.930
IL8 (pg/mL)	10 025.50 (3560.10‐28232.51)	4752.38 (3701.52‐6100.95)	.053
CXCL10 (pg/mL)	321.48 (102.40‐1009.22)	751.18 (546.47‐1032.59)	.076
CXCL11 (pg/mL)	15.17 (4.99‐46.09)	49.30 (32.32‐75.22)	.052
CCL2 (pg/mL)	365.94 (216.12‐619.63)	443.28 (354.67‐553.96)	.544
CCL3 (pg/mL)	119.26 (47.71‐298.08)	57.46 (44.69‐73.87)	.053
CCL4 (pg/mL)	1066.69 (463.22‐2456.38)	815.37 (613.44‐1083.77)	.516
CCL5 (pg/mL)	10.87 (4.76‐24.80)	7.40 (5.76‐9.50)	.295
CCL13 (pg/mL)	12.72 (6.06‐26.72)	29.49 (23.79‐36.56)	.009
CCL17 (pg/mL)	18.39 (7.89‐42.85)	32.83 (24.74‐43.57)	.159
CCL26 (pg/mL)	4.40 (1.52‐12.73)	8.39 (5.76‐12.21)	.249
TNFα (pg/mL)	40.37 (9.95‐163.74)	4.62 (3.29‐6.51)	<.001
TNFR1 (pg/mL)	1443.14 (683.72‐3046.09)	855.34 (693.80‐1054.49)	.096
TNFR2 (pg/mL)	654.95 (276.11‐1553.58)	389.65 (308.09‐492.80)	.141
VEGF (pg/mL)	1344.23 (918.23‐1967.88)	1666.02 (1450.95‐1912.97)	.283

Note

Data are presented as geometric means, with 95% CI.

### 3.3 Microbiome clustering within disease group

To ascertain whether the clusters observed in the combined analysis were driven predominantly by one disease group, model‐based cluster analysis was performed in asthma and COPD individually. In both severe asthma and COPD, two clusters were observed at phylum level, broadly reflecting the clusters observed in the combined analysis. Distribution of phyla and genera across the clusters are demonstrated in Figures S2 and S3.

In both asthma and COPD, Cluster 1 was smaller with predominance of Proteobacteria and increased proportions of *Haemophilus* and *Moraxella.* In COPD but not asthma, this cluster demonstrated increased proportions of *Pseudomonas*. In both conditions, Cluster 2 was characterized by predominance of Firmicutes and an increased proportion of *Streptococcus* spp.

There was a lack of distinguishing clinical characteristics between the microbiome clusters in both conditions. In severe asthma, Cluster 1 was older and in COPD, they demonstrated an increased sputum neutrophilia. γP:F ratio was significantly higher in Cluster 1 in both diseases, as was the sputum IL1β concentration.

### 3.4 γP:F ratio can be used to identify the *Haemophilus*‐high subgroup

**Figure 5:**
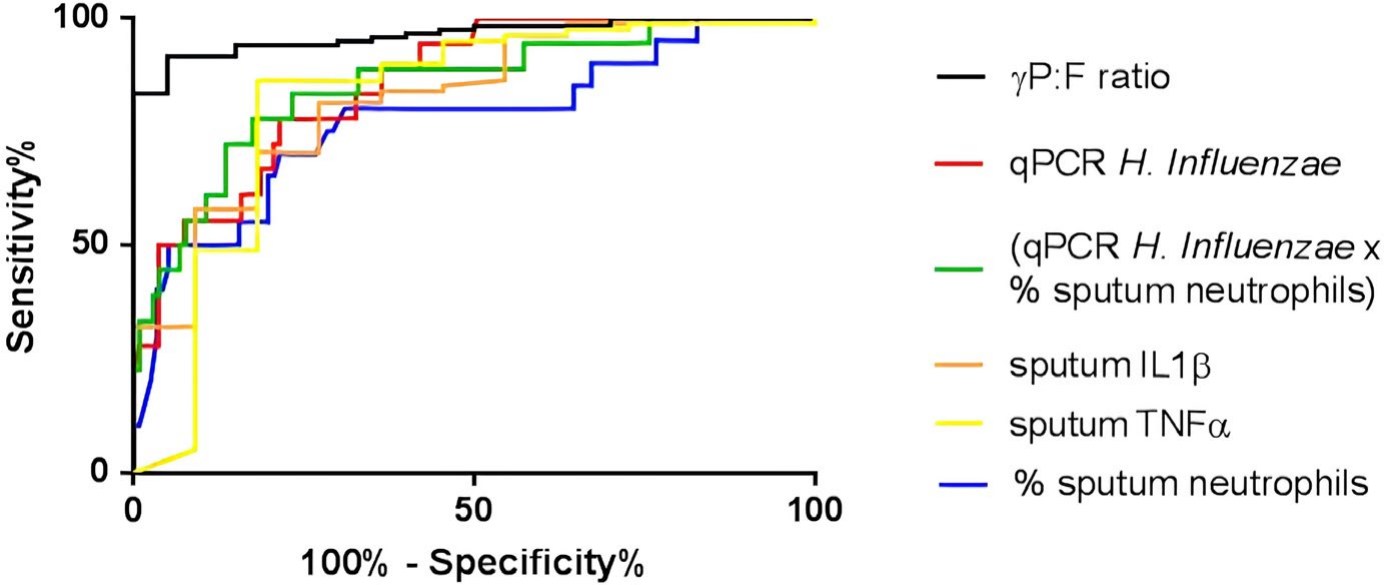
ROC analysis to determine predictors of *Haemophilus*‐high vs. *Haemophilus*‐low subgroup

Measurable characteristics differentiating the subgroups were reviewed as potential biomarkers. ROC analysis to determine the best biomarkers of the *Haemophilus*‐high versus *Haemophilus*‐low subgroups was undertaken between qPCR *H* *infuenzae* (copies/ml), % sputum neutrophils, γP:F ratio and sputum IL1β and TNFα concentration (Figure 5). γP:F ratio was most predictive of the *Haemophilus*‐high group with AUC of 0.96 (*P* < .0001); however, copies/mL of *H influenzae* as measured by qPCR was also strongly predictive with AUC 0.86 (*P* < .0001). Combining these markers with the % sputum neutrophils did not increase the predictive value. The AUC for both IL1β and TNFα was 0.82 (*P* < .001).

Twenty‐two subjects across both airways disease had serial samples with qPCR *H influenzae* measurements at stable state without an intervening exacerbation event. The intra‐class correlation was 0.53 demonstrating moderate repeatability. We dichotomised this group of subjects using the previously described threshold *H influenzae *>10^8^ copies/mL^16^ and found that all of the seven subjects above this threshold at baseline had persistently elevated *H influenzae* in subsequent samples, whereas in the subjects in the lower *H influenzae* group 6/16 switched groups.

## 4 DISCUSSION

In this study, we identified two airway disease sputum microbiome clusters in stable severe asthma and COPD, differentiated by predominance of the genus *Haemophilus*. Consistent with our hypothesis, these *Haemophilus*‐high and *Haemophilus*‐low clusters were discriminated by the γP:F ratio. Specific qPCR for *H Influenzae* demonstrated a clear separation between the clusters in terms of high and low absolute abundance. Interestingly, there were no clinical characteristics associated with the *Haemophilus*‐high cluster; however, these subjects had raised levels of the pro‐inflammatory mediators sputum IL1β and TNFα. Analysis comparing asthma to COPD demonstrated no significant difference in the major phyla groups overall; however, similar subgroups, characterized by high and low *Haemophilus* and γP:F ratios, were identifiable within disease groups analysed individually. These findings indicate that the *Haemophilus*‐high and *Haemophilus*‐low clusters are not disease specific.

Here, we have applied topological data analysis in concert with cluster analysis for the first time to explore the airway microbiome in asthma and COPD. Classical clustering methods such as Hierarchical or K‐Means split a data set apart and can result in data points being artificially separated. Topological data analysis does not split a data set apart, but rather provides a two‐dimensional representation of the original high‐dimensional data set, retaining the essential features of the underlying geometry of the original data. The combination of topological and cluster analysis strengthens our assertion that there are two sputum microbiome clusters best described as *Haemophilus*‐high and *Haemophilus*‐low.

Identification of two microbiome sputum clusters in airways disease, *Haemophilus*‐high and *Haemophilus*‐low, at clinical stability is consistent with previous findings at exacerbations.^16^ The *Haemophilus*‐high cluster was associated with increased pro‐inflammatory mediators IL1β and TNFα in asthma and COPD. Clinical trials targeting these cytokines in asthma or COPD have failed to demonstrate efficacy and with anti‐TNFα have led to increased adverse events suggesting that targeting inflammation in this group is unlikely to be a successful strategy. However, previously reported response to the macrolide azithromycin in moderate‐to‐severe asthma was associated with positive bacterial culture,^13^ and with reductions in *Haemophilus influenzae* in the overall population.^28^ Our findings therefore suggest that the *Haemophilus‐*high cluster, although likely to represent a limited proportion of the total airways disease population, might represent an important subgroup of patients with airways disease in whom bacterial dysbiosis plays an important role and is potentially amenable to anti‐microbial interventions.

The development of well‐validated, widely accessible, relevant biomarkers is integral to implementation of targeted therapies towards precision medicine in the clinic. We found that the γP:F ratio was the most sensitive and specific biomarker within our data set to identify the *Haemophilus*‐high cluster. In this study, the γP:F ratio was determined from the microbiomic analysis; however, our previous work demonstrates that γP:F ratio can be derived using qPCR analysis.^15^ These molecular approaches are able to provide near‐patient, rapid diagnostic tests that can be applied clinically. Whether this strategy identifies the subgroup of patients with airways disease that could benefit from antibiotics or, with increasing concerns regarding antibiotic stewardship, alternative anti‐microbial strategies such as vaccination targeting *H influenzae*, oral probiotics acting via the gut‐lung axis or bacteriophage therapy warrants further investigation. Importantly, although the repeatability of the sputum *H influenzae* levels in those with the very highest values was good suggesting some phenotype stability, some subjects do change phenotype perhaps highlighting the need for repeated measures of biomarkers of airway ecology in the clinic.

Our study has a number of potential limitations. The sample size was good for the analysis of asthma and COPD but the combined clusters were small, as were clusters within the individual diseases and therefore limits our confidence in these comparisons. The majority of sputum samples tested were spontaneous rather than induced samples, and data from both approaches were combined. Sputum induction was undertaken only if an inadequate spontaneous sample was produced. We have previously shown that the microbiome from these approaches are comparable and therefore do not anticipate this will have affected our interpretations,^19^ although we recognize that differences in sampling technique impact microbiomic analysis and require consideration when interpreting data. Additionally, analysis of sputum has an acquisition bias as it excludes those that fail to produce a sputum sample. This is a minor issue for COPD with successful sputum production in >90% of subjects^29^ but with success up to 90%^30^ in asthma means that clusters cannot be generalized to all asthmatics. Likewise, the number of subjects that had repeated sampling adequate for molecular microbiological analysis was small and limits our ability to study phenotypic stability. We did not explore the sensitivity and specificity of routine culture to identify the *Haemophilus*‐high group in our analysis; however, it is well established that molecular techniques are more sensitive for assessment of the complete microbial profile. Additionally, our study did not include detailed radiological evaluation for bronchiectasis, which commonly co‐exists with asthma and COPD; therefore, it is unclear whether co‐morbid bronchiectasis is associated with one of the sputum microbiome clusters. Finally, the airway microbiome is a complex, dynamic ecological system and, whilst we suggest that more severe airflow obstruction observable in the *Haemophilus*‐high group is driven by disease, it is not possible to exclude reverse causality, whereby the airway conditions in subjects with airways disease permit colonization by *Haemophilus* species.^31^ The effect of environmental factors, including allergens, and competition between organisms colonizing the airway^32^ on microbiome composition warrants further study in future.

In summary, we have identified two sputum microbiome clusters, *Haemophilus*‐high and *Haemophilus*‐low, in airways disease. These clusters can be distinguished by the sputum γP:F ratio or *H influenzae*, which present opportunities for rapid molecular biomarkers in the clinic to determine cluster membership. Whether identification of these microbiome clusters and more specifically the *Haemophilus*‐high cluster is associated with a favourable response to antibiotic or nonantibiotic anti‐microbial interventions in airways disease needs to be tested in future trials.

## CONFLICTS OF INTEREST

SD, MRi, KH, MAG, MRa and MBar have nothing to declare. DD reports personal fees from AstraZeneca, Boehringer Ingelheim and Chiesi, outside the submitted work; MBaf reports personal fees from AstraZeneca, Boehringer Ingelheim, Chiesi, GlaxoSmithKline, Novartis and Pfizer, outside the submitted work; ESC, PN and PR are employees of AstraZeneca, which supported the study; IDP reports personal fees and nonfinancial support from AstraZeneca and Boehringer Ingelheim, personal fees from Aerocrine, Almirall, Novartis, GlaxoSmithKline, Genentech, Regeneron, Merck & Co., Schering‐Plough, Mylan Speciality (Dey Pharma), Napp Pharmaceuticals and Respivert, outside the submitted work; RDM and LR are former employees of AstraZeneca, which supported the study; CEB has received grants and personal fees paid to his institution from AstraZeneca, GlaxoSmithKline, Roche/Genentech, Novartis, Chiesi, Pfizer, Teva, Sanofi/Regeneron, Glenmark, Mologic, PreP and Vectura, outside the submitted work.

## AUTHORS’ CONTRIBUTION

SD, MRi and MAG undertook the data analysis and statistical analysis. DD and MBaf undertook patient recruitment, data collection and were involved in data analysis. KH and MRa were involved in microbiologic assessment. ESC, PN, PR and LR were involved in sputum mediator assessment and analysis. IDP was co‐supervisor for the COPD patients. MBar was involved in microbiologic assessment. CEB and RDM led the design of the study, data collection, data interpretation, data analysis and had full access to the data and were responsible for the integrity of the data and final decision to submit. All authors contributed to the study design, writing of the manuscript and have approved the final version for submission.
