# Supplementary material for: Sputum microbiomic clustering in asthma and chronic obstructive pulmonary disease reveals a Haemophilus‐predominant subgroup
Source: Allergy. 2019 Oct 21;75(4):808–17. doi: 10.1111/all.14058 (PMC7217013; doi:10.1111/all.14058)

**Sputum microbiomic clustering in asthma and COPD reveals a *Haemophilus*-predominant subgroup**

Sarah Diver, MBChB^1^, Matt Richardson, PhD^1^, Koirobi Haldar, PhD^1^, Michael A Ghebre, PhD^2^, Mohammadali Yavari Ramsheh, MSc^1^, Mona Bafadhel, PhD^3^, Dhananjay Desai, PhD^1^, E. Suzanne Cohen, PhD^4^, Paul Newbold, PhD^5^, Laura Rapley, PhD^6^, Paul Rugman, PhD^4^, Ian D. Pavord, MD^3^, Richard D. May, PhD^7^, Michael Barer, PhD^1^, Christopher. E. Brightling, PhD^1^

**^1^**Institute for Lung Health, NIHR Leicester Biomedical Research Centre, Department of Respiratory Sciences, College of Life Sciences, University of Leicester and University Hospitals of Leicester NHS Trust, Leicester, UK

^2^hVIVO Services Limited; 42 New Rd, Whitechapel, London, UK

^3^Respiratory Medicine Unit, Nuffield Department of Medicine, NDM Research Building, Old Road Campus, University of Oxford, Oxford, UK

^4^AstraZeneca, Milstein Building, Granta Park, Cambridge, UK

^5^AstraZeneca, One MedImmune Way, Gaithersburg, Maryland, USA

^6^GlaxoSmithKline, Gunnels Wood Road, Stevenage, UK

^7^Sosei Heptares, Granta Park, Cambridge, UK

**Correspondence to:**

Professor CE Brightling^1^

Institute for Lung Health, University Hospitals of Leicester

Groby Road, Leicester, LE3 9QP, UK

Telephone: +44 116 258 3998 E-mail: [ceb17@le.ac.uk](mailto:ceb17@le.ac.uk)

Table S1. Clinical characteristics for disease groups within ecological clusters.

| **Characteristic** | **Cluster 1** | | **Cluster 2** | |  |
| --- | --- | --- | --- | --- | --- |
|  | **Asthmatic patients (n = 5)** | **Patients with COPD (n = 15)** | **Asthmatic patients (n = 58)** | **Patients with COPD (n = 63)** |  |
| **Male sex, n (%)** | 2 (40.0) | 12 (80.0) | 32 (55.2) | 47 (74.6) ≠ |  |
| **Age*** | 60.6 (0.7) | 68.8 (2.3) | 54.8 (1.6) | 67.8 (1.2) ≠ | ** |
| **Duration of disease^¥^** | 26.0 (6.5 to 39.5) | 9.3 (6.2 to 16.4) | 31.5 (10.3 to 50.0) | 5.3 (3.6 to 11.6) ≠ |  |
| **BMI^*^** | 28.0 (3.2) | 27.5 (1.2) | 29.4 (0.9) | 26.1 (0.6) ≠ |  |
| **Current/ex-smokers, n (%)** | 2 (40.0) | 14 (93.3) | 23 (39.7) | 62 (98.4) ≠ |  |
| **Pack year history^¥¶^** | 10.3 (7.5 to 13.0) | 39.5 (20.8 to 53.0) ≠ | 10.0 (2.0 to 21.4) | 48.5 (36.7 to 64.3) ≠ |  |
| **Exacerbations in the last year^¥^** | 1.5 (1.0 to 4.3) | 3.0 (1.3 to 7.0) | 3.0 (2.0 to 4.0) | 2.5 (1.0 to 4.0) |  |
| **Maintenance prednisolone, n (%)** | 0 (0.0) | 4 (26.7) | 33 (57.9) | 3 (5.8) ≠ | **# |
| **Daily prednisolone dose^¥$^** | NA | 5.0 (4.3 to 5.0) | 10.0 (7.5 to 12.5) | 5.0 (consistent) ≠ |  |
| **Daily inhaled corticosteroid dose^¥^** | 2000 (900 to 2000) | 1800 (700 to 2000) | 1600 (1000 to 2000) | 1400 (800 to 2000) |  |
| **Pre-FEV_1_ (L) ^*^** | 1.65 (0.08) | 1.26 (0.14) ≠ | 2.11 (0.09) | 1.19 (0.06) ≠ | ** |
| **Pre-FEV_1_ (% predicted) ^*^** | 62.62 (7.79) | 41.78 (3.98) ≠ | 72.93 (2.84) | 41.29 (2.15) ≠ |  |
| **Post-FEV_1_ (L) ^*^** | 1.74 (0.12) | 1.27 (0.15) | 2.32 (0.10) | 1.25 (0.07) ≠ | ** |
| **Post-FEV_1_ (% predicted) ^*^** | 65.92 (8.69) | 42.51 (4.33) ≠ | 79.14 (2.81) | 43.61 (2.28) ≠ |  |
| **FEV_1_/FVC ratio^*^** | 0.63 (0.06) | 0.48 (0.03) ≠ | 0.68 (0.02) | 0.45 (0.02) ≠ |  |
| **VAS, cough (mm) ^¥^** | 37.0 (10.0 to 65.0) | 73.0 (40.0 to 88.0) | 35.0 (8.5 to 53.5) | 50.0 (17.3 to 67.3) | # |
| **VAS, dyspnoea (mm) ^¥^** | 30.0 (11.5 to 64.5) | 68.0 (48.0 to 71.0) | 30.0 (10.0 to 54.5) | 57.0 (33.3 to 69.0) ≠ |  |
| **Blood neutrophils (x 10^9^/L) ^*^** | 4.30 (0.70) | 6.45 (0.78) | 5.81 (0.29) | 5.41 (0.20) |  |
| **Blood eosinophils (x 10^9^/L)** | 0.40 (0.11 to 1.51) | 0.23 (0.20 to 0.26) | 0.25 (0.19 to 0.33) | 0.21 (0.18 to 0.25) |  |
| **Sputum neutrophils count (%)^¥^** | 61.50 (54.63 to 94.50) | 94.25 (85.50 to 96.50) | 68.25 (47.00 to 83.25) | 69.25 (52.50 to 85.00) | # |
| **Sputum eosinophil count (%)** | 3.57 (0.58 to 21.92) | 0.73 (0.39 to 1.36) ≠ | 1.78 (1.09 to 2.91) | 1.35 (0.96 to 1.91) |  |
| **γP:F ratio** | 16.67 (5.18 to 53.60) | 12.20 (4.81 to 30.92) | 0.21 (0.14 to 0.31) | 0.14 (0.08 to 0.25) | **# |

#Data are presented as geometric means, with 95% CI, unless otherwise stated. *Mean (SEM). ¥Median (1st and 3rd quartiles). ¶ Pack year history of current and ex-smokers. $Dose for those patients prescribed oral corticosteroids. ≠Significant difference between asthma and COPD within cluster. **, #Overall significant difference between clusters in asthma and COPD respectively. Abbreviations: COPD=Chronic Obstructive Pulmonary Disease; BMI=Body Mass Index; FEV_1_=Forced Expiratory Volume in the First Second; FVC=Forced Vital Capacity; VAS=Visual Analogue Scale; γP:F ratio=γProteobacteria to Firmicutes ratio.

Table S2. Sputum mediators for disease groups within identified ecological clusters.

| **Sputum mediator** | **Cluster 1** | | **Cluster 2 (n=121)** | |  |
| --- | --- | --- | --- | --- | --- |
|  | **Asthmatic patients**  **(n = 5)** | **Patients with COPD**  **(n = 15)** | **Asthmatic patients**  **(n = 58)** | **Patients with COPD**  **(n = 63)** |  |
| **IL1β (pg/mL)** | 461.10 (30.12 to 7058.41) | 551.65 (169.39 to 1796.56) | 79.00 (55.27 to 112.94) | 54.28 (29.77 to 98.98) | **# |
| **IL5 (pg/mL)** | 1.97 (0.18 to 21.23) | 2.39 (0.39 to 14.77) | 4.60 (2.94 to 7.20) | 1.16 (0.58 to 2.35) ≠ |  |
| **IL6 (pg/mL)** | 28.47 (2.03 to 399.65) | 800.51 (155.61 to 4118.20) ≠ | 74.51 (50.62 to 109.67) | 528.03 (307.60 to 906.40) ≠ |  |
| **IL6R (pg/mL)** | 249.12 (50.02 to 1240.58) | 289.56 (132.88 to 631.00) | 296.23 (232.28 to 377.78) | 160.92 (110.00 to 235.41) ≠ |  |
| **IL8 (pg/mL)** | 8184.4 (450.9 to 148547.0) | 14237.6 (8860.6 to 22877.4) | 4699.8 (3482.7 to 6342.2) | 4159.4 (2483.6 to 6966.0) |  |
| **CXCL10 (pg/mL)** | 460.17 (36.37 to 5821.41) | 290.50 (41.03 to 2056.72) | 936.89 (641.60 to 1368.07) | 489.99 (263.53 to 911.07) ≠ |  |
| **CXCL11 (pg/mL)** | 24.44 (2.91 to 205.53) | 13.58 (1.72 to 106.99) | 65.24 (39.61 to 107.43) | 24.80 (10.93 to 56.29) ≠ |  |
| **CCL2 (pg/mL)** | 253.78 (79.24 to 812.75) | 539.19 (273.30 to 1063.76) | 378.09 (290.93 to 491.37) | 720.47 (475.95 to 1090.63) ≠ |  |
| **CCL3 (pg/mL)** | 56.81 (11.64 to 277.14) | 299.13 (82.47 to 1085.00) | 48.61 (35.41 to 66.72) | 83.46 (56.25 to 123.83) ≠ | # |
| **CCL4 (pg/mL)** | 627.29 (84.58 to 4652.52) | 1995.69 (957.46 to 4159.72) | 705.06 (484.68 to 1025.65) | 1170.25 (774.00 to 1769.38) |  |
| **CCL5 (pg/mL)** | 13.44 (1.96 to 92.28) | 11.23 (3.39 to 37.19) | 9.96 (7.38 to 13.43) | 3.69 (2.56 to 5.32) ≠ | # |
| **CCL13 (pg/mL)** | 13.12 (5.38 to 32.01) | 19.69 (5.96 to 65.12) | 26.43 (20.53 to 34.03) | 36.51 (23.21 to 57.43) | # |
| **CCL17 (pg/mL)** | 20.18 (3.71 to 109.65) | 26.87 (10.98 to 65.75) | 39.35 (28.18 to 54.95) | 22.31 (12.55 to 39.68) |  |
| **CCL26 (pg/mL)** | 6.93 (0.57 to 84.13) | 2.79 (1.26 to 6.17) | 13.89 (9.36 to 20.62) | 2.52 (1.26 to 5.01) ≠ |  |
| **TNFα (pg/mL)** | 16.53 (0.55 to 496.45) | 111.52 (32.84 to 378.71) | 4.22 (2.84 to 6.29) | 5.61 (2.66 to 11.81) | # |
| **TNFR1 (pg/mL)** | 963.57 (163.88 to 5665.63) | 2380.51 (1006.81 to 5628.48) | 717.23 (557.24 to 923.16) | 1252.09 (887.98 to 1765.49) ≠ |  |
| **TNFR2 (pg/mL)** | 410.85 (48.34 to 3491.98) | 1059.11 (404.68 to 2771.86) | 380.34 (283.49 to 510.29) | 404.17 (261.97 to 623.56) |  |
| **VEGF (pg/mL)** | 1139.9 (644.0 to 2017.8) | 1857.3 (972.4 to 3547.5) | 1828.4 (1530.9 to 2183.6) | 1320.3 (1065.2 to 1636.5) ≠ |  |

Data are presented as geometric means, with 95% CI, unless otherwise stated. ≠Significant difference between asthma and COPD within cluster. **, #Overall significant difference between clusters in asthma and COPD respectively.

Figure S1***.*** Alpha diversity measures compared between; a) asthma and COPD disease groups, and b) the *Haemophilus*-high (HH) and *Haemophilus*-low (HL) ecological clusters.


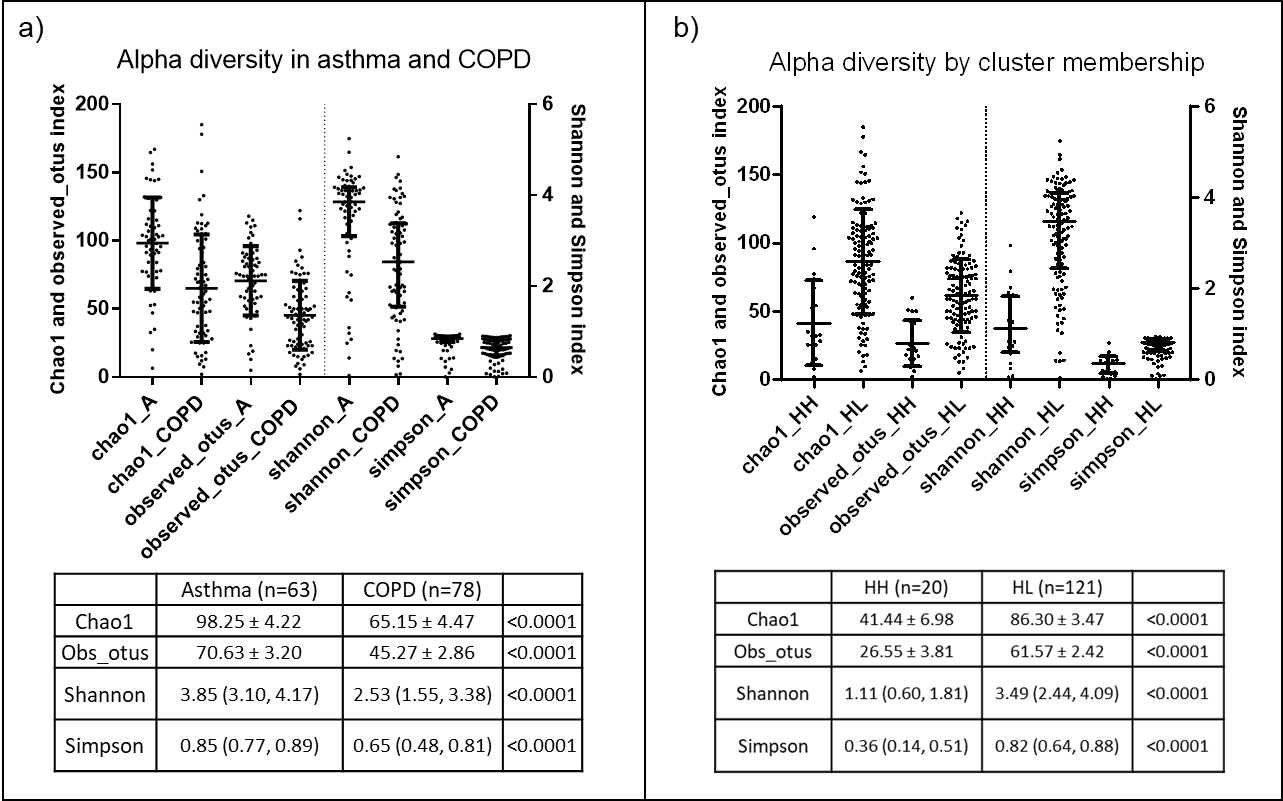


Figure S2. Figure S2 shows results of Topological data analysis for combined microbiological data (OTU proportions) from asthmatic and COPD subjects at genus level, networks were coloured by; a) *Haemophilus*, b) disease group (Asthma or COPD), c) *Streptococcus*, d) *Rothia,* e) *Actinomyces* and f) *Prevotella*.


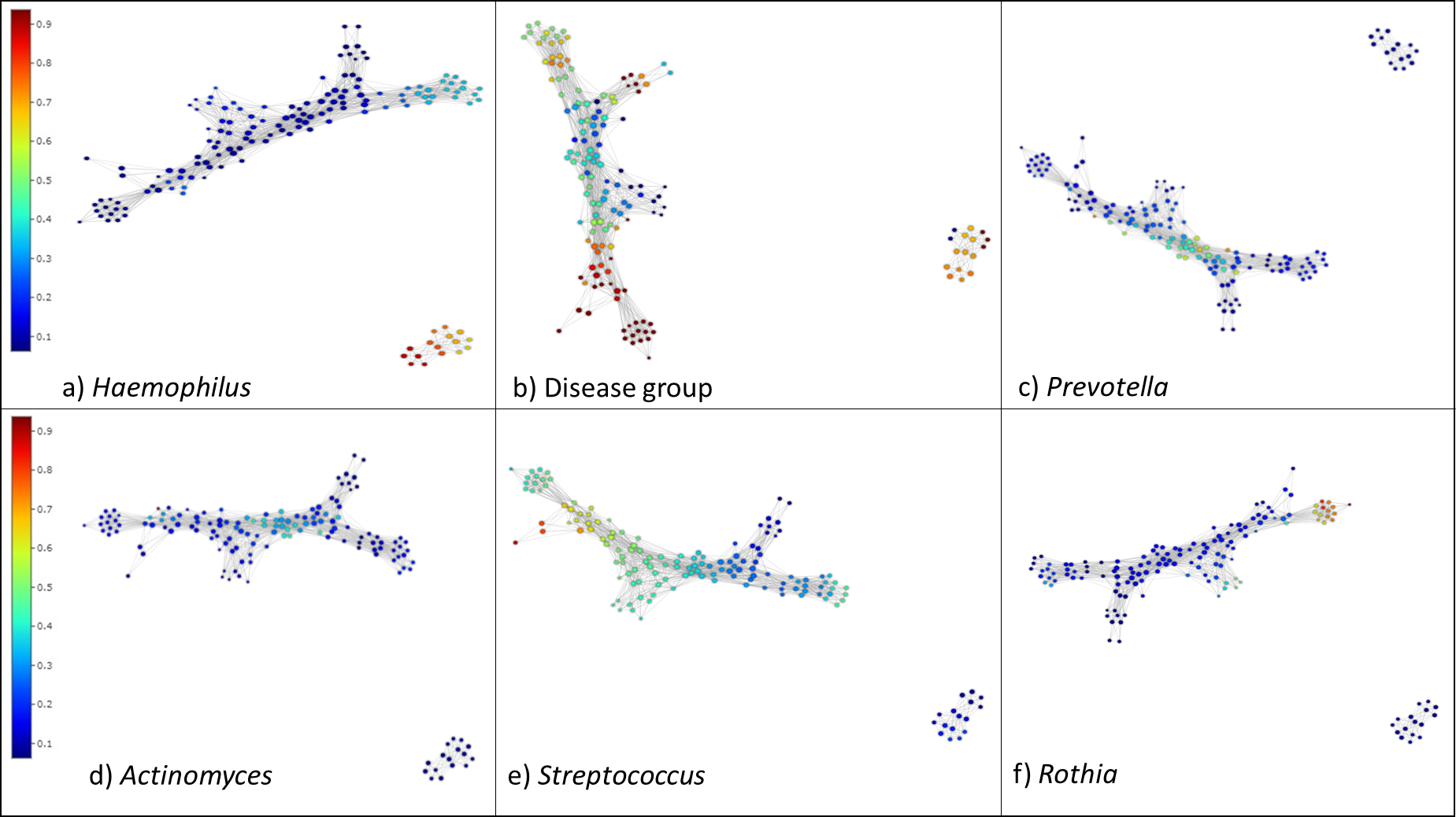


Figure S3. Proportions of organisms belonging to; a) the 5 most abundant phyla, and b) abundant or important genera across the identified ecological clusters of severe asthma and moderate-to-severe COPD. Statistically significant differences between clusters within disease group are indicated by * for asthma, ^ for COPD.


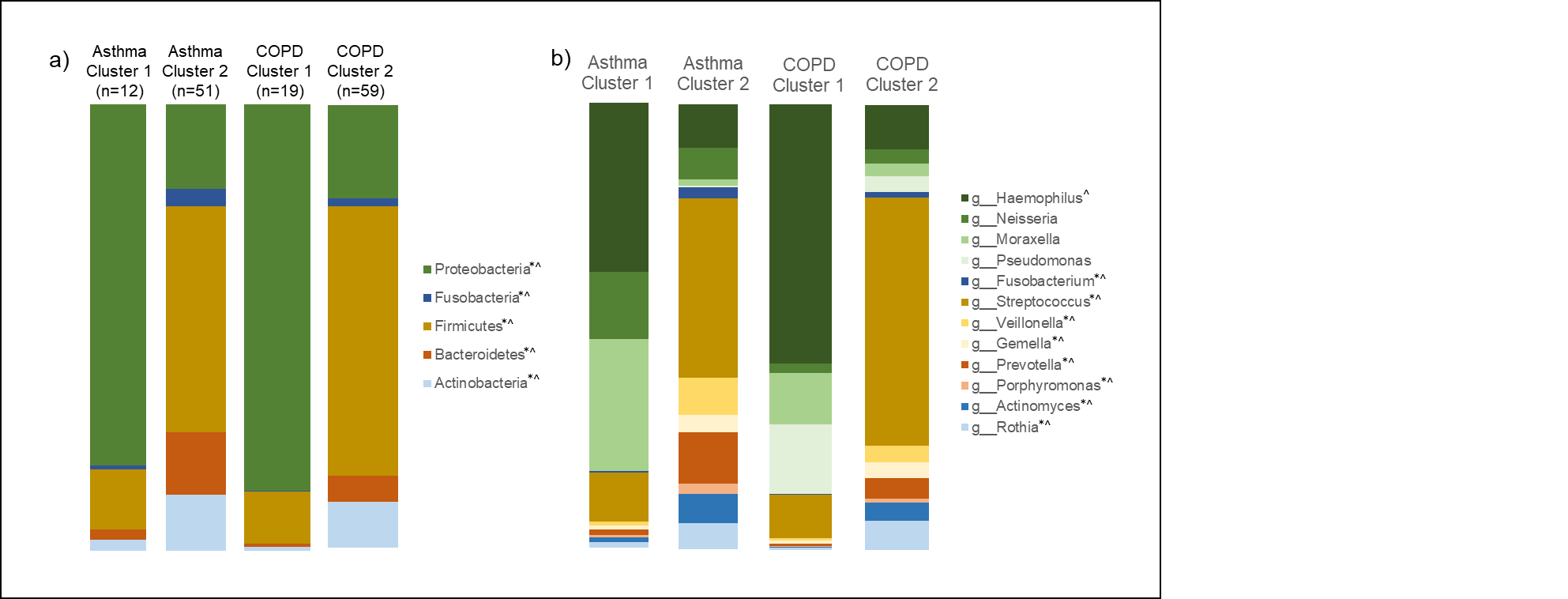

Supplement: Supplementary file 1 [file ALL-75-808-s001.docx]
